# Growth inhibitory, immunosuppressive, cytotoxic, and genotoxic effects of γ-terpinene on *Zeugodacus cucurbitae* (Coquillett) (Diptera: Tephritidae)

**DOI:** 10.1038/s41598-023-43499-8

**Published:** 2023-09-30

**Authors:** Diksha, Sumit Singh, Evani Mahajan, Satwinder Kaur Sohal

**Affiliations:** Department of Zoology, Guru Nanak Dev University, Amritsar, Punjab 143005 India

**Keywords:** Entomology, Physiology

## Abstract

γ-Terpinene, a monoterpene widely present in essential oils of many medicinal and aromatic plants with numerous biological properties, was evaluated for its insecticidal activity against melon fruit fly, *Zeugodacus cucurbitae* (Coquillett). Different concentrations (5, 25, 125, 625, and 3125 ppm) of γ-terpinene along with control were fed to larvae of melon fly. The number of pupae formed and adults emerged declined significantly after treatment. Morphologically deformed adults and pupae were also observed. The developmental duration too prolonged in treated larvae. Food assimilated, mean relative growth rate, larval weight gain, and pupal weight also declined. In the larvae treated with LC_30_ and LC_50_ concentrations, there was a decline in the titers of phenoloxidase and total hemocyte count, and variations were observed in the differential hemocyte count, suggesting an immunosuppressive effect of γ-terpinene on melon fly. Both concentrations also led to an increase in the apoptotic and necrotic cells as well as decrease in the viable hemocytes in the circulating hemolymph of treated larvae. Comet parameters (tail length, % tail DNA, tail moment, and olive tail moment) of γ-terpinene fed larvae increased significantly. Given the observed effects of γ-terpinene on normal developmental and nutritional physiology, its immunosuppressive properties, and its potential for genome damage, it can be considered for incorporation into integrated pest management strategies for controlling *Z. cucurbitae*.

## Introduction

For the management of insect pests, chemically synthesized pesticides are being extensively used^1^. Also, the widespread use of these pesticides has certain negative repercussions such as human health hazards, toxicity to ecologically beneficial organisms, water, soil, and air pollution, and development of resistance in pests^2,3^. Biopesticides, therefore, have grabbed the attention of scientific community owing to their low toxicity and biodegradability^4^ with one such being the botanicals or plant-based pesticides. Botanicals are known to exhibit diverse biological activities against arthropods such as insecticidal, acaricidal, antifeedant, repellent, and anti-ovipositional effects^5^. They act as toxins by intervening in the normal vital functioning of the insect body and are known to affect the insect at various levels, including morphological, physiological, genomic, and immunological^6,7^. Many plant allelochemicals act as antifeedants and deterrents, thereby reducing their consumption by the insect which ultimately impacts their growth and leads to morphological deformities in the insect pest^8,9^. Some phytochemicals are known to generate oxidative free radicals and these deleterious free radicals when excessively generated damage the biomolecules of insect pest including its DNA which ultimately results in cell death^10^.

The innate immune system of insects plays an important role in defending the individual from foreign agents and includes a cascade of specific and non-specific responses^11^. Hemocytes are known to participate in coagulation, encapsulation, and phagocytosis^12^, whereas the key enzyme of an insect’s immune system, phenoloxidase is responsible for melanogenesis^11^. The number and proportions of various hemocytes are also important for insects to develop environmental fitness^13^. Due to this highly efficient immune system, many insect pests are able to flourish in agroecosystems and cause economic losses. Moreover, there exists a cross-talk between immune system and nervous system of insects^12^. In insects, metamorphosis and development is regulated by ecdysteroids. They also regulate immunological functions such as the conversion of immunocytes to phagocytic cells and the release of hemocytes from hematopoietic organs^14^. Hemocytes, on the other hand, are also known to regulate metamorphosis^15^. Any disruption in the endocrine functioning of insects therefore can hamper immunological and developmental physiology. A morphologically deformed/weak and immunocompromised pest will have reduced chances of survival in the field, as it will be exposed to several other natural control agents. Consequently, plant-based compounds with growth inhibitory, immunomodulatory, and cyto-genotoxic effects can be potent candidates for incorporation into integrated pest management programs.

Terpenes form a major group of secondary metabolites produced by the plants. Amongst them, monoterpenes are main components of many plants and in some essential oils account for almost 90% of the oil^16^ and are the most successful group of botanicals^17^. They are known to show various properties such as anticancer, anti-inflammatory, antidiabetic, antioxidant, and antihypertensive^18^. Monoterpenes are derived from universal C_5_ building blocks, DMAPP (dimethylallyl diphosphate) and IPP (isopentenyl diphosphate). In plants, these key precursors can be synthesized by two compartmentalized pathways, the mevalonate (MVA) pathway that operates in the cytosol and the 2-C-methyl-D-erythritol-4-phosphate (MEP) pathway which occurs in the plastids^19,20^. Further, they combine to form a C_10_ moiety having two isoprene units that can be arranged into acyclic and cyclic structures^21^. γ-Terpinene (1-Isopropyl-4-methyl-1,4-cyclohexadiene) is a monoterpene found in various plant species such as *Thymus vulgaris* L.^22^, *Eucalyptus camaldulensis* Dehnh.^23^, *Nigella sativa* L., *Cuminum cyminum* L.^24^, *Majorana hortensis* Moench^25^, *Protium icicariba* (DC.) Marchand, *Citrus deliciosa* Tenore, *Origanum onites* L.^26^, *Melissa officinalis* L.^27^, *Satureja thymbra* L.^28^, and *Pistacia khinjuk* Stocks^29^, and is known for its antibacterial^22,30,31^, antifungal^22,31^, acaricidal^28^, antileshmanial^32^, antinociceptive^26^, antioxidant^33^, anticancer^31^, antiviral^34^, and insecticidal^25,27,35^ activities.

The melon fruit fly, *Zeugodacus cucurbitae* (Coquillett) (Diptera: Tephritidae) (formerly known as *Bactrocera cucurbitae*) is a serious agroeconomic pest known to attack many varieties of vegetables and fruit crops^6,36,37^ and has a wide geographical distribution including Asia, Australia-Oceania, Africa, Hawaii, and South America^37,38^. *Z. cucurbitae* is subject of many quarantine, detection, exclusion, and eradication protocols owing to its high invasibility, vast adaptability, and high reproductive potential^39^. *Z. cucurbitae* has a wide host range with *Momordica charantia* L., *Cucumis melo* L., *Cucumis melo* L. var*. momordica* (Roxb.), *Trichosanthes anguina* L., *Cucumis sativus* L., and *Luffa acutangula* (L.) Roxb. being the preferred hosts^6,37^. Maggots of melon fly feed voraciously and cause serious damage to the crop thereby leading to economic loss. The damage caused to fruit due to melon fly attack has been reported to range from 30 to 100%^6,37^ and particularly in India this loss ranges from 40 to 60%^6^. Despite a large number of studies on the insecticidal effects of terpenes against pest insects, few studies have been carried out to evaluate the insecticidal effects of monoterpene rich essential oils on pestiferous fruit flies^40–48^. Moreover, there exists a lacuna on how plant-based chemicals impact the immune system of these fruit flies. Therefore, this study was envisaged to evaluate the insecticidal effect of γ-terpinene against *Z. cucurbitae* and to highlight the post-ingestive toxicity of γ-terpinene by investigating nutritional parameters, cellular and humoral immune system, viability of hemocytes, and DNA damage of *Z. cucurbitae*.

## Results

### Effect on growth and development

**Figure 1:**
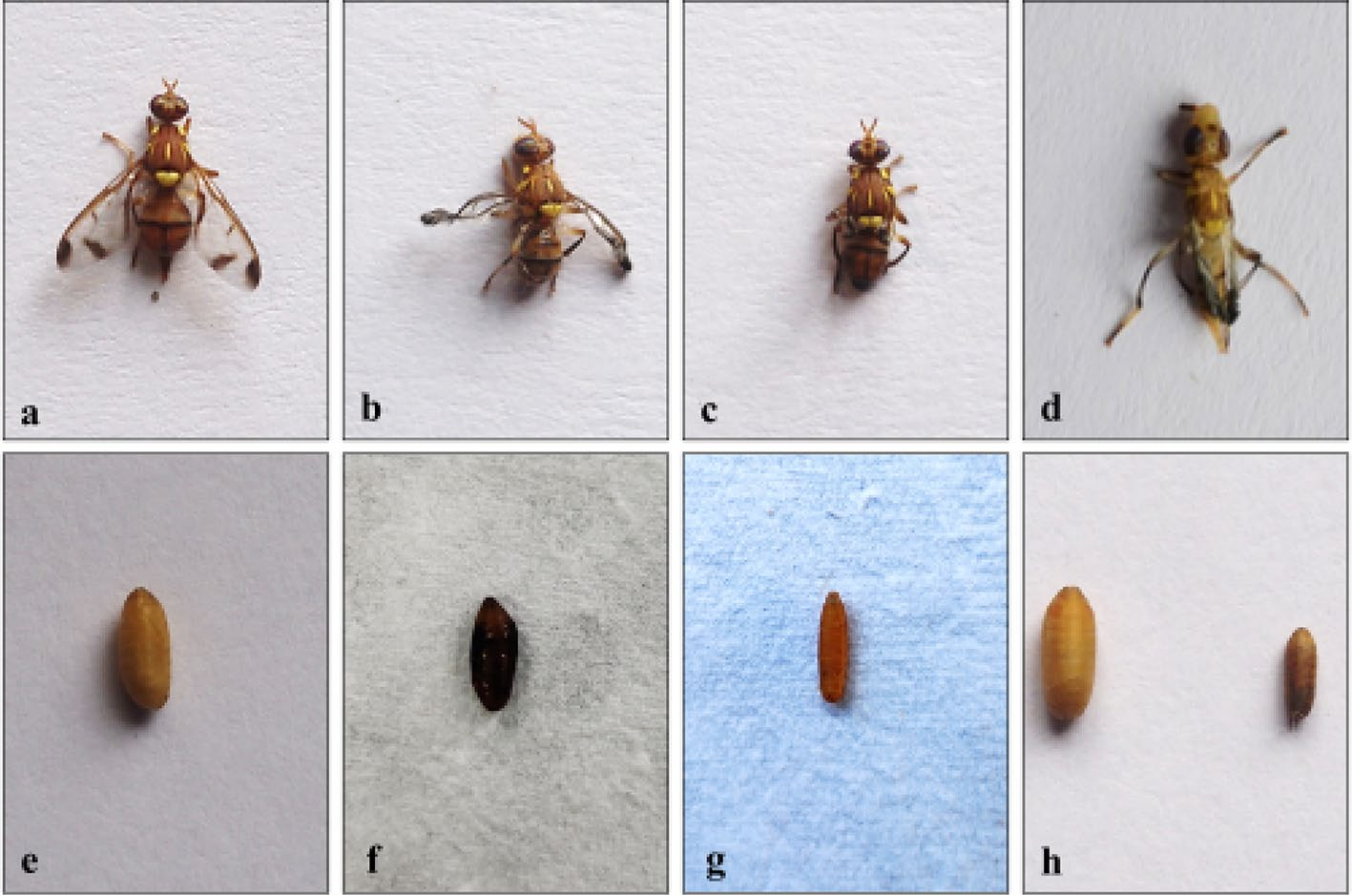
Morphological deformities observed after treatment of *Z. cucurbitae* with γ-terpinene. Normal adult (**a**), deformed adults (**b–d**), normal pupa (**e**), deformed pupae (**f,g**), reduced size of pupa as compared to normal pupa (**h**).

Our results for bioassays reveal an impairing impact of γ-terpinene on the normal developmental processes of *Z. cucurbitae.* All three larval instars when treated with the increasing concentrations of γ-terpinene showed a significant decline in the number of pupae formed (Table 1). Maximum decline (88.61% as compared to control) in the number of pupae formed was observed in case of second instar larvae at 3125 ppm concentration followed by first instar larvae (85.37% compared to control) and third instar larvae (41.47% compared to control). The LC_30_ and LC_50_ values for second instar larvae were computed to be 90.39 ppm and 1066 ppm, respectively. The percentage of adults emerged also declined significantly in a concentration-dependent manner in all three larval instars when treated with increasing concentrations of γ-terpinene (p ≤ 0.01). Maximum decline was observed in case of first and second instar larvae, where at 3125 ppm concentration, it declined by 92.42% and 92.75% compared to that in control, respectively (Table 1). Furthermore, the adults emerged and pupae formed from treated larvae also showed some morphological deformations (Fig. 1).

**Table 1:** Pupation (%) and Adult emergence (%) of different larval instars of *Z. cucurbitae* when fed on artificial diet amended with different concentrations of γ-terpinene.

Concentration (ppm)	Pupation (%)			Adult emergence (%)		
	First instar	Second instar	Third instar	First instar	Second instar	Third instar
Control	91.11 ± 4.10^e^	87.78 ± 3.18^d^	91.11 ± 3.30^bc^	73.33 ± 3.85^c^	76.67 ± 3.75^d^	78.89 ± 2.05^c^
5	76.67 ± 2.85^de^	76.67 ± 2.85^cd^	92.22 ± 3.18^c^	67.78 ± 3.18^c^	68.89 ± 3.30^d^	72.22 ± 3.18^c^
25	71.11 ± 2.81^cd^	71.11 ± 2.81^c^	86.67 ± 3.85^bc^	62.22 ± 2.22^c^	52.22 ± 2.05^c^	63.33 ± 4.47^bc^
125	57.78 ± 5.35^bc^	54.44 ± 4.69^b^	86.67 ± 3.85^bc^	44.44 ± 3.30^b^	31.11 ± 5.07^b^	53.33 ± 3.85^b^
625	50.00 ± 3.75^b^	51.11 ± 5.07^b^	76.67 ± 2.85^b^	37.78 ± 3.30^b^	22.67 ± 3.69^b^	51.11 ± 6.13^b^
3125	13.33 ± 3.85^a^	10.00 ± 2.85^a^	53.33 ± 3.85^a^	5.56 ± 2.68^a^	5.56 ± 2.05^a^	32.22 ± 3.18^a^
F-value	48.49**	55.19**	17.56**	64.37**	63.54**	17.35**

**Indicates Significant at 1% level of significance. Values are Mean ± SE. Mean values within a column sharing the same superscript letter are not significantly different according to Tukey’s test at p ≤ 0.05.

**Figure 2:**
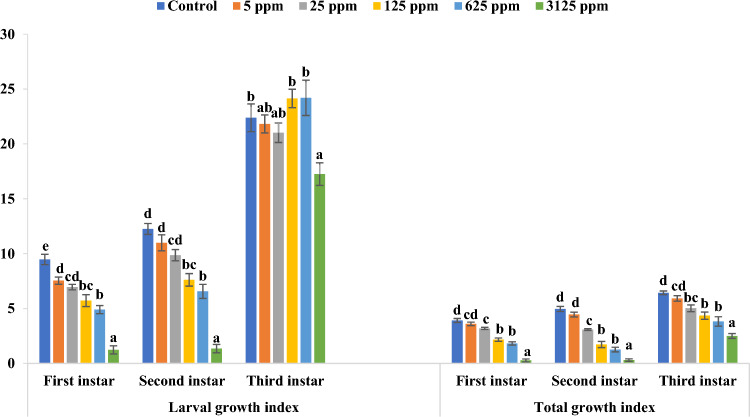
Larval growth index and Total growth index of different larval instars of *Z. cucurbitae* when fed on artificial diet amended with different concentrations of γ-terpinene. Bars represent Mean ± SE. Bars sharing the same letter are not significantly different according to Tukey’s test at p ≤ 0.05.

γ-Terpinene also led to an overall prolongation in the developmental durations (larval period, pupal period, and total development period) of melon fly larvae (Table 2). The larval period prolonged significantly in case of first instar larvae (p ≤ 0.01). Maximum increase was found at 3125 ppm where the larvae took 1.40 days more than control to metamorphose into pupae. There was a non-significant increase in the larval period of γ-terpinene treated second instar larvae. On the contrary, the larval period for third instar larvae declined as compared to control. Pupal and total development period also increased significantly for all three larval instars (p ≤ 0.01). The pupal and total development period increased maximally in the treatment of second instar larvae, where the pupal period increased by 3.06 days while the total development period increased by 3.29 days as compared to control at 3125 ppm. The larval growth index and total growth index of all three larval instars declined significantly after γ-terpinene treatment (p ≤ 0.01) (Fig. 2). Maximum reduction in larval and total growth index was found at 3125 ppm in case of second instar larvae, where they declined by 88.98% and 93.95% when compared to control, respectively.

**Table 2:** Larval period (days), pupal period (days), and total development period (days) of different larval instars of *Z. cucurbitae* when fed on artificial diet amended with different concentrations of γ-terpinene.

Concentration (ppm)	Larval period (days)			Pupal period (days)			Total development period (days)		
	First instar	Second instar	Third instar	First instar	Second instar	Third instar	First instar	Second instar	Third instar
Control	9.65 ± 0.22^a^	7.18 ± 0.10^a^	4.11 ± 0.16^b^	9.09 ± 0.31^a^	8.29 ± 0.14^a^	8.16 ± 0.25^a^	18.74 ± 0.21^a^	15.46 ± 0.12^a^	12.26 ± 0.12^ab^
5	10.19 ± 0.09^a^	7.07 ± 0.31^a^	4.23 ± 0.05^b^	8.65 ± 0.21^a^	8.44 ± 0.34^a^	7.96 ± 0.12^a^	18.84 ± 0.23^a^	15.50 ± 0.14^a^	12.19 ± 0.10^a^
25	10.23 ± 0.18^a^	7.26 ± 0.26^a^	4.12 ± 0.06^b^	9.27 ± 0.33^a^	9.65 ± 0.41^ab^	8.48 ± 0.13^a^	19.50 ± 0.27^a^	16.91 ± 0.30^b^	12.60 ± 0.10^ab^
125	10.13 ± 0.15^a^	7.13 ± 0.11^a^	3.60 ± 0.13^a^	10.47 ± 0.21^b^	10.95 ± 0.18^bc^	8.68 ± 0.24^a^	20.60 ± 0.18^b^	18.08 ± 0.11^c^	12.28 ± 0.15^ab^
625	10.20 ± 0.03^a^	7.80 ± 0.16^a^	3.20 ± 0.11^a^	10.50 ± 0.26^b^	10.41 ± 0.41^bc^	10.11 ± 0.14^b^	20.71 ± 0.26^b^	18.21 ± 0.40^c^	13.31 ± 0.16^c^
3125	11.05 ± 0.23^b^	7.40 ± 0.24^a^	3.10 ± 0.16^a^	9.75 ± 0.19^ab^	11.35 ± 0.31^c^	9.75 ± 0.24^b^	20.50 ± 0.22^b^	18.75 ± 0.16^c^	12.85 ± 0.24^bc^
F-value	7.52**	NS	17.80**	8.57**	16.38**	20.54**	15.41**	37.68**	8.34**

Values are Mean ± SE. Mean values within a column sharing the same superscript letter are not significantly different according to Tukey’s test at p ≤ 0.05.

*NS* non-significant.

**Indicates Significant at 1% level of significance.

### Effect on pupal weight

**Figure 3:**
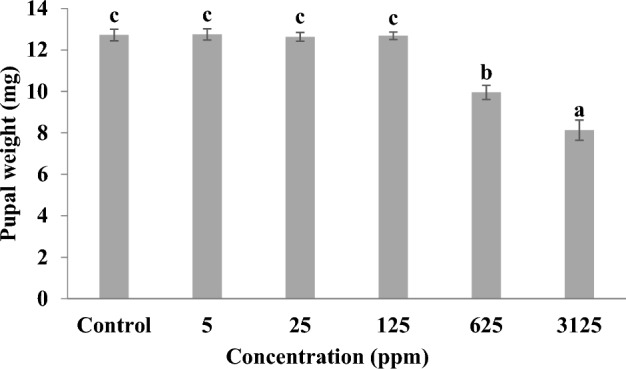
Pupal weight (mg) of *Z. cucurbitae* when second instar (64–72 h old) larvae were fed on artificial diet amended with different concentrations of γ-terpinene. Bars represent Mean ± SE. Bars sharing the same letter are not significantly different according to Tukey’s test at p ≤ 0.05.

The reduced weight of pupae also supported our findings for growth inhibitory effects of γ-terpinene on melon fly (Fig. 3). Pupal weight (mg) declined significantly after γ-terpinene treatment (F-value = 40.17; p ≤ 0.01) with maximum decline (36.08%) observed at 3125 ppm concentration as compared to control.

### Effect on nutritional parameters

Nutritional alterations were also observed in γ-terpinene fed second instar larvae (Table 3) (p ≤ 0.01). There was a significant reduction in the larval weight gain with γ-terpinene treatment. Maximum decline in larval weight gain was observed at highest concentration of 3125 ppm where weight gain per larva decreased from 10.24 mg in control to 3.65 mg with treatment. Food assimilated (FA) with respect to control also declined maximally at 3125 ppm concentration. A similar declining trend was also seen for mean relative growth rate (MRGR). There was a 52.83% reduction in MRGR at highest concentration of 3125 ppm as compared to control.

**Table 3:** Nutritional parameters viz. larval weight gain, food assimilated (FA) with respect to control, and mean relative growth rate (MRGR) of *Z. cucurbitae* when second instar larvae (64–72 h old) were fed on artificial diet amended with different concentrations of γ-terpinene.

Concentration (ppm)	Larval weight gain (mg)	FA w.r.t. control (mg)	MRGR (mg/mg/day)
Control	10.24 ± 0.26^d^	-	0.53 ± 0.02^d^
5	9.77 ± 0.29^d^	20.09 ± 0.34^d^	0.52 ± 0.02^d^
25	7.98 ± 0.37^c^	18.53 ± 0.27^cd^	0.43 ± 0.01^c^
125	7.07 ± 0.39^bc^	17.78 ± 0.32^bc^	0.41 ± 0.02^bc^
625	5.66 ± 0.41^b^	16.45 ± 0.52^b^	0.33 ± 0.01^b^
3125	3.65 ± 0.34^a^	13.93 ± 0.53^a^	0.25 ± 0.02^a^
F-value	51.92**	31.81**	36.20**

Values are Mean ± SE. Mean values within a column sharing the same superscript letter are not significantly different according to Tukey’s test at p ≤ 0.05.

**Indicates Significant at 1% level of significance.

### Immune response

#### Effect on activity of phenoloxidase

**Figure 4:**
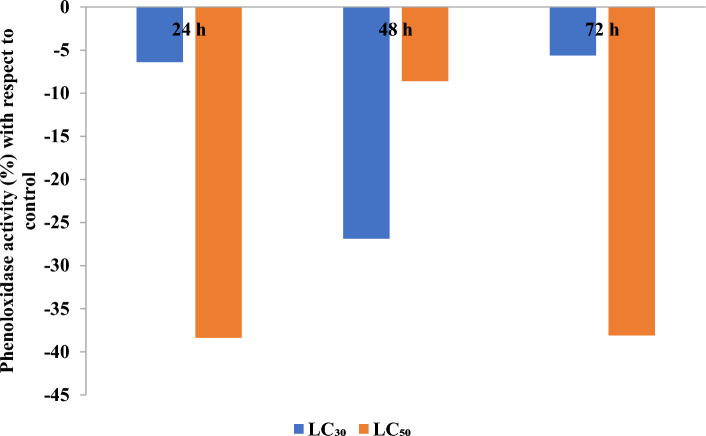
Phenoloxidase activity (% with respect to control) of *Z. cucurbitae* when second instar (64–72 h old) larvae were fed on artificial diet amended with LC_30_ and LC_50_ concentrations of γ-terpinene.

Phenoloxidase activity declined at all three treatment intervals at both LC_30_ and LC_50_ concentrations (Fig. 4). Maximum decline in enzyme activity was observed with LC_50_ concentration after 24 h of treatment, where it declined by 38.38% with respect to control.

#### Effect on total hemocyte count (THC)

γ-Terpinene treatment led to a significant decline in the total hemocyte count of *Z. cucurbitae* second instar larvae as compared to control larvae (p ≤ 0.01) (Table 4). The decline in total hemocyte count was considerably greater at LC_50_ than at LC_30_ after 24 and 72 h of treatment. However, at 48 h treatment interval, the decline in total hemocyte count was more at LC_30_ than at LC_50_ concentration as compared to control.

**Table 4:** Total hemocyte count (THC) (Cells/mm^3^) of *Z. cucurbitae* when second instar larvae (64–72 h old) were fed on artificial diet amended with LC_30_ and LC_50_ concentrations of γ-terpinene.

Concentration (ppm)	Total hemocyte count/mm^3^		
	24 h	48 h	72 h
Control	7685.00 ± 220.99^b^	9052.50 ± 178.01^c^	6404.17 ± 168.84^b^
LC_30_	7525.83 ± 114.65^b^	3954.17 ± 296.04^a^	5681.67 ± 300.61^b^
LC_50_	3481.67 ± 258.60^a^	5030.83 ± 261.18^b^	3620.00 ± 237.54^a^
F-value	132.12**	115.51**	35.72**

Values are Mean ± SE. Mean values within a column sharing the same superscript letter are not significantly different according to Tukey’s test at p ≤ 0.05.

**Indicates Significant at 1% level of significance.

#### Effect on differential hemocyte count (DHC)

**Figure 5:**
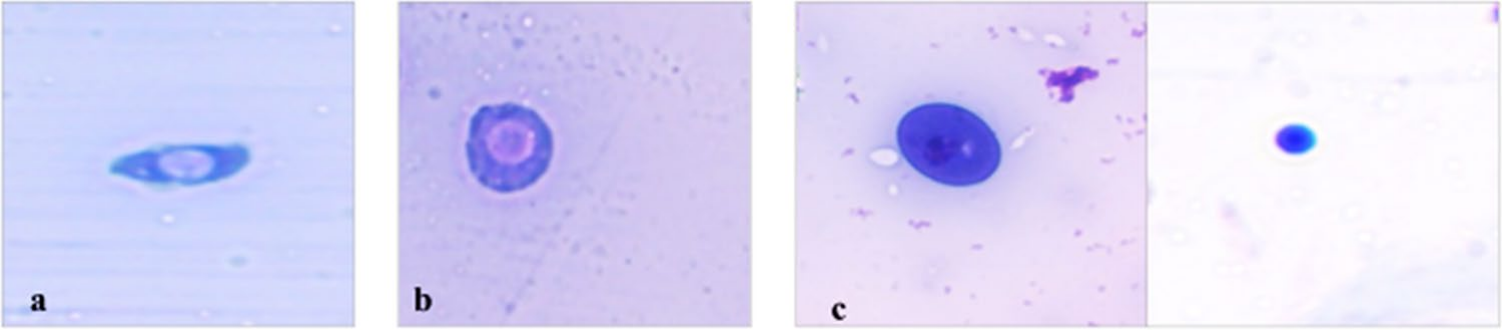
Various hemocytes of *Z. cucurbitae*. Plasmatocyte (**a**), Granulocyte (**b**), and hemocytes that were collectively labelled as Others (**c**).

**Figure 6:**
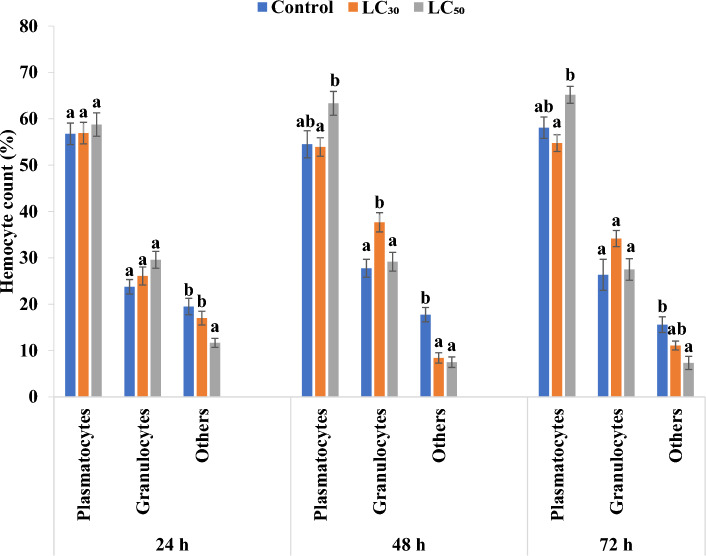
Differential hemocyte count of *Z. cucurbitae* when second instar larvae (64–72 h old) were fed on artificial diet amended with LC_30_ and LC_50_ concentrations of γ-terpinene. Bars represent Mean ± SE. Bars sharing the same letter are not significantly different according to Tukey’s test at p ≤ 0.05.

Hemocytes were identified on the basis of morphological features described by Gupta^49^. Plasmatocytes are pleiomorphic in shape, mostly spindle shaped (Fig. 5a). Granulocytes, on the other hand are usually round in shape, with cytoplasm rich in granules (Fig. 5b). Plasmatocytes and granulocytes were identified and rest of the hemocytes were collectively labelled as others (Fig. 5c). The proportion of plasmatocytes increased in larvae treated with LC_50_ concentration of γ-terpinene as compared to control larvae (Fig. 6). Maximum percentage of granulocytes was observed with LC_30_ concentration of γ-terpinene after 48 h of treatment where they reached 37.67% in comparison to 27.75% in control (F-value = 7.15; p ≤ 0.01) (Fig. 6). The population of other hemocytes declined at all the treatment intervals i.e., at 24 h (F-value = 7.65; p ≤ 0.01), 48 h (F-value = 19.68; p ≤ 0.01), and 72 h (F-value = 8.89; p ≤ 0.01) with both the lethal concentrations of γ-terpinene (Fig. 6).

### Cytotoxic effects

**Figure 7:**
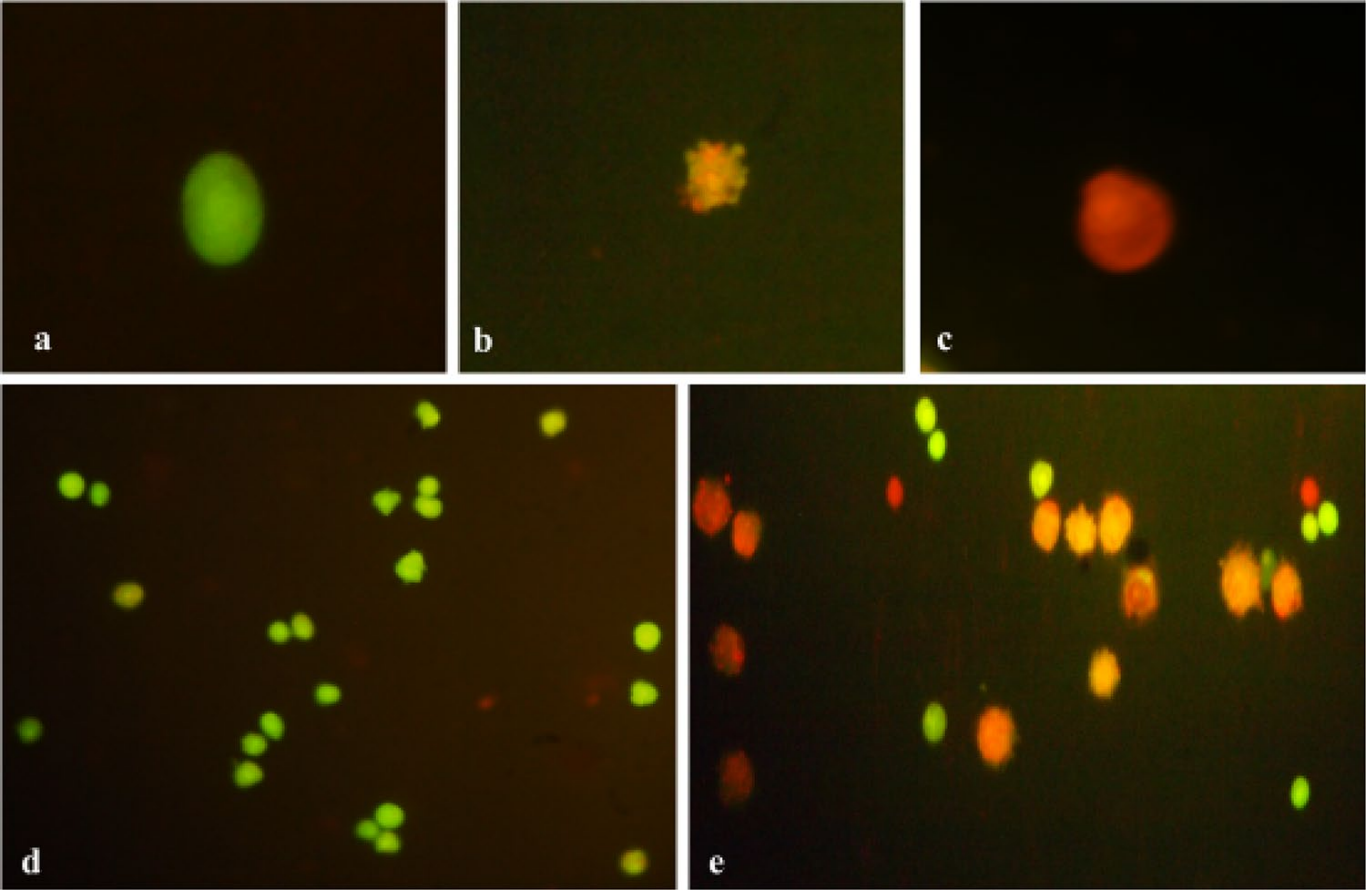
Dual Acridine orange (AO)/Ethidium bromide (EtBr) staining of *Z. cucurbitae* hemocytes. Viable/living cell (**a**), Apoptotic cell (**b**), Necrotic cell (**c**), hemocytes of control larvae (**d**), hemocytes of γ-terpinene treated larvae (**e**).

Hemocytes were identified as viable, apoptotic, and necrotic using acridine orange/ethidium bromide (AO/EtBr) dual staining (Fig. 7). The frequency of viable cells decreased significantly at all three treatment intervals with both LC_30_ and LC_50_ concentrations of γ-terpinene (p ≤ 0.01) (Table 5). Maximum decline in viability of hemocytes was observed after 72 h of treatment with LC_50_ concentration. When compared to control, maximum increase in the percentage of apoptotic and necrotic cells was found after 24 h and 72 h of treatment, respectively with LC_50_ concentration (p ≤ 0.01) (Table 5).

**Table 5:** γ-Terpinene induced variations in viable, apoptotic, and necrotic cells in hemolymph of *Z. cucurbitae* when second instar larvae (64–72 h old) were fed on artificial diet amended with LC_30_ and LC_50_ concentration.

Treatment interval	Concentration (ppm)	Viable cells (%)	Apoptotic cells (%)	Necrotic cells (%)
24 h	Control	88.78 ± 0.55^c^	8.28 ± 0.45^a^	2.94 ± 0.20^a^
	LC_30_	69.28 ± 1.16^b^	23.83 ± 1.01^b^	6.89 ± 0.72^b^
	LC_50_	55.94 ± 1.44^a^	35.56 ± 1.27^c^	8.50 ± 0.87^b^
	F-value	219.77**	198.09**	18.71**
48 h	Control	86.89 ± 0.57^b^	9.61 ± 0.39^a^	3.50 ± 0.31^a^
	LC_30_	58.06 ± 1.27^a^	30.56 ± 1.19^b^	11.39 ± 0.80^b^
	LC_50_	58.39 ± 1.56^a^	32.50 ± 0.89^b^	9.11 ± 1.09^b^
	F-value	187.26**	205.88**	25.50**
72 h	Control	85.50 ± 0.51^c^	9.94 ± 0.48^a^	4.56 ± 0.29^a^
	LC_30_	65.78 ± 1.23^b^	25.67 ± 0.82^b^	8.56 ± 1.19^b^
	LC_50_	46.89 ± 1.50^a^	35.67 ± 1.00^c^	17.44 ± 1.15^c^
	F-value	277.47**	264.53**	46.39**

Values are Mean ± SE. Mean values sharing the same superscript letter are not significantly different according to Tukey’s test at p ≤ 0.05.

**Indicates significant at 1% level of significance.

### Genotoxic effects

**Figure 8:**
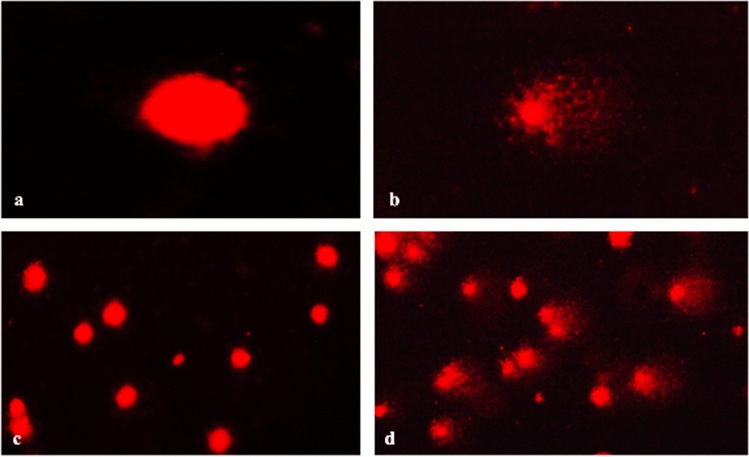
γ-Terpinene induced DNA damage in hemocytes of *Z. cucurbitae.* DNA of control larvae (**a**,**c**) and γ-terpinene treated larvae (**b**,**d**).

Genotoxic effects of γ-terpinene were observed on hemocytes of second instar larvae of melon fly larvae (Fig. 8). All the comet parameters viz. tail length (µm), % tail DNA, tail moment (TM), and olive tail moment (OTM) increased significantly with both LC_30_ and LC_50_ concentrations of γ-terpinene. Maximum increase in tail length was observed with LC_50_ concentration (p ≤ 0.01) (Table 6). A similar increase was also perceived in other comet parameters with LC_50_ being more damaging to the DNA as compared to LC_30_ of γ-terpinene (p ≤ 0.05) (Table 6).

**Table 6:** Alterations in all comet parameters viz. tail length (µm), % tail DNA, tail moment (TM), olive tail moment (OTM) obtained from hemocytes of *Z. cucurbitae* when second instar larvae (64–72 h old) were fed on artificial diet amended with LC_30_ and LC_50_ concentrations of γ-terpinene.

Treatment interval	Concentration (ppm)	Tail length (µm)	% Tail DNA	Tail moment (TM)	Olive tail moment (OTM)
24 h	Control	9.77 ± 0.48^a^	2.01 ± 0.27^a^	0.26 ± 0.04^a^	1.10 ± 0.14^a^
	LC_30_	28.29 ± 2.23^b^	10.53 ± 1.43^b^	4.18 ± 0.56^b^	6.17 ± 0.68^b^
	LC_50_	44.01 ± 0.85^c^	13.08 ± 1.22^b^	6.24 ± 0.45^c^	7.44 ± 0.40^b^
	F-value	148.72**	27.87**	54.35**	53.09**
48 h	Control	10.11 ± 0.74^a^	2.37 ± 0.70^a^	0.42 ± 0.12^a^	1.39 ± 0.61^a^
	LC_30_	24.67 ± 1.82^b^	9.81 ± 1.63^b^	4.00 ± 0.87^b^	5.86 ± 0.85^b^
	LC_50_	36.65 ± 2.36^c^	11.30 ± 2.24^b^	4.77 ± 1.05^b^	7.16 ± 0.58^b^
	F-value	56.04**	8.38*	8.68*	19.29**
72 h	Control	11.09 ± 0.60^a^	2.91 ± 0.10^a^	0.48 ± 0.04^a^	1.41 ± 0.20^a^
	LC_30_	31.83 ± 1.41^b^	13.03 ± 0.68^b^	5.95 ± 0.08^b^	8.55 ± 0.51^b^
	LC_50_	44.11 ± 1.85^c^	15.07 ± 2.38^b^	7.19 ± 0.96^b^	10.22 ± 0.36^c^
	F-value	144.95**	20.76**	40.80**	154.59**

Values are Mean ± SE. Mean values sharing the same superscript letter are not significantly different according to Tukey’s test at p ≤ 0.05.

**Indicates significant at 1% level of significance.

*Indicates Significant at 5% level of significance.

## Discussion

γ-Terpinene showed an adverse impact on the growth and development of *Z. cucurbitae*. There was a drastic reduction in the number of pupae formed from treated larvae as most larvae failed to reach pupation. Insecticidal effects of γ-terpinene have been previously reported by Gong and Ren^35^ against cotton bollworm, *Helicoverpa armigera* (Hübner). Jiang et al.^50^ too reported larvicidal effects of γ-terpinene from *Litsea cubeba* (Lour.) Pers. against third instar larvae of cabbage looper, *Trichoplusia ni* (Hübner). γ-Terpinene was also reported to show strong toxicity against red flour beetle, *Tribolium castaneum* (Herbst) and cigarette beetle, *Lasioderma serricorne* (Fabricius)^51^. Toxic effects of γ-terpinene have also been demonstrated against confused flour beetle, *Tribolium confusum* Jacquelin Du Val and Mediterranean flour moth, *Ephestia kuehniella* (Zeller)^52^. Similarly, Rizzo et al.^46^ reported insecticidal potential of essential oils of *Thymbra spicata* L., *Ocimum gratissimum* L., *Pimpinella anisum* L., and *Trachyspermum ammi* (L.) Sprague rich in monoterpenes thymol, p-cymene, γ-terpinene, (*E*)-anethole, and carvacrol against adults of olive fruit fly, *Bactrocera oleae* (Rossi). Basij et al.^53^ too documented larvicidal activity of γ-terpinene and other monoterpenes (thymol and p-cymene) from *Carum copticum* L. against Asiatic rice borer, *Chilo suppressalis* Walker. Abdelgaleil et al.^17^ attributed the lethality of monoterpenes to their neurotoxic effects on AChE, GABA, octopamine receptors, voltage-gated sodium channels, and glutamate-gated chloride channels of the target pest.

Our results also showed failure of treated larvae to metamorphose into pupae, decline in the percentage of adults emerged, deformed pupae and adults, and decline in pupal weight. There was prolongation of developmental duration i.e., larval, pupal, and total development periods. The larval and total growth indices of the larvae also declined drastically. Similar results were observed by Abdelgaleil et al.^54^. They found Egyptian cotton leafworm, *Spodoptera littoralis* (Boisduval) larvae when treated with various monoterpenes, phenylpropenes, and sesquiterpenes showed an increase in developmental duration (larval and pupal duration), reduction in pupation percentage, decline in adults emerged, and reduction of pupal weight. Our results for growth inhibitory activities of γ-terpinene are supported by another study conducted by Abdelgaleil et al.^55^, where they reported terpenes and phenylpropenes to inhibit the growth of *S. littoralis* larvae. The decrease in larval growth index after treatment with *Melaleuca alternifolia* (Maiden and Betche) Cheel and its main constituents, terpinene-4-ol and γ-terpinene in *S. littoralis* larvae was also observed by Ismail et al.^56^. El-Minshawy et al.^44^ also reported reduction in pupation and adult emergence when second instar larvae of peach fruit fly, *Bactrocera zonata* (Saunders) were treated with monoterpenes viz. (*R*)-camphor, (*R*)-carvone, and (1*R*, 2*S*, 5*R*) menthol. Further, deformities in the adults emerged were also observed. Ecdysteroids in insects regulate the normal development and metamorphosis^14^, so the latent effects of γ-terpinene on melon fly could be the result of its interference with the endocrine system which might have altered its normal functioning.

The post-ingestive toxic effects of γ-terpinene on the larvae of *Z. cucurbitae* were evident from the altered nutritional parameters i.e., decreased larval weight gain, food assimilated with respect to control, and mean relative growth rate. Ismail et al.^56^ also reported that *M. alternifolia* and its main constituents, terpinene-4-ol and γ-terpinene significantly reduced the feeding efficiency of *S. littoralis* larvae. They too observed a decline in weight gain and relative growth rate of the larvae. Chen et al.^57^ also observed anti-nutritional effect of carvacrol, a monoterpenoid phenol on gypsy moth, *Lymantria dispar* (Linnaeus) larvae. When fed on diet containing carvacrol, there was a significant reduction in weight gain of the larvae and decrease in food intake compared to control larvae. Similarly, the weight and size of *S. littoralis* larvae were found to be reduced after treatment of terpenoids (γ-terpinene, p-cymene, and carvacrol) from *Origanum vulgare* L. and leaf discs of *O. vulgare* itself. The consumption of food was also reduced in treated larvae as compared to control larvae^58^. Several histological studies have revealed that terpenoids can cause dysfunction of the insect gut. Gut epithelial degeneration and necrosis in yellow fever mosquito, *Aedes aegypti* (Linnaeus) has been observed by Pintong et al.^59^ after treatment with terpenoid rich essential oils of *Ageratum conyzoides* L.

Insects, when infected with foreign agents, protect themselves with the help of highly coordinated cellular and humoral immune cascades. Plasmatocytes, granulocytes, prohemocytes, spherulocytes, and oenocytoids are the most commonly reported insect hemocytes in literature^12^. Hemocyte-mediated responses such as phagocytosis, nodulation, and encapsulation constitute the cellular defense. Whereas, humoral defense includes the production of antimicrobial peptides, ROS, RNS, and enzymes that regulate coagulation and melanization of hemolymph^11,12^. Phenoloxidase forms a major component of humoral immunity and plays a crucial role in wound healing, sclerotization, and melanization. It is kept in zymogen form and upon activation by the biological activators, is converted locally into phenoloxidase. Quinones generated by the phenoloxidase may lethally act against foreign agents^60^. A suppression in the titers of phenoloxidase in *Z. cucurbitae* larvae was found after γ-terpinene treatment at all three treatment intervals and with both LC_30_ and LC_50_ concentrations. Similarly, terpinene-4-ol has also been reported to inhibit phenoloxidase activity in fifth instar larvae of northern armyworm, *Mythimna separata* Walker^61^. Mahajan et al.^7^ reported similar suppression in the activity of phenoloxidase upon β-caryophyllene treatment to tobacco cutworm, *Spodoptera litura* (Fabricius) larvae. Hemocytes are well known to be the source of phenoloxidase^11^, therefore decline in the level of phenoloxidase titers can be due to fall in the number of circulating hemocytes. Our results for the total hemocyte count of the *Z. cucurbitae* larvae also depicted a decline in the number of circulating hemocytes at both LC_30_ and LC_50_ concentrations. Several studies support this finding whereby many plant products, essential oils, or growth regulators have been reported to influence the count of circulating hemocytes^7,62–65^. For example, essential oils isolated from four ecotypes of *C. cyminum* having cuminaldehyde, γ-terpinene, p-cymene, β-pinene, and α-phellandrene as main components, reduced the hemocyte count of pink stem borer, *Sesamia cretica* Lederer^66^. Botanicals are known to interfere with the normal hematopoiesis and lead to decreased cell division thereby reducing the number of circulating hemocytes. They can also influence ecdysteroids that regulate the release of hemocytes into hemolymph from hematopoietic organs^67,68^. γ-Terpinene led immunological challenge also elicited changes in the proportions of circulating hemocyte types in *Z. cucurbitae* larvae. There was an increase in the proportion of plasmatocytes and granulocytes. On the other hand, a decrease in other hemocytes was observed. Many botanicals have been reported to cause alterations in the types of circulating hemocytes in the hemolymph of insect^13,66–70^. A review of previously reported literature reveals that dynamism in the proportions of hemocyte types i.e., change in the number of one hemocyte kind and simultaneous increase in the number of another hemocyte can be attributed to the acquirement of desirable immunological function (e.g., melanisation and/ phagocytosis). Moreover, their proportions also vary throughout different life-cycle stages and throughout different larval instars^64^. Plasmatocytes and granulocytes population in melon fruit fly larvae might have been increased in response to γ-terpinene stress as they are the key hemocyte types participating in hemocyte-mediated responses^65,71^ and decline in other hemocyte types may be to compensate the disrupted immunological functions by proliferating into one or the other type of hemocytes.

Furthermore, hemocytes of insects due to their multi-faceted roles are considered more sensitive to external and internal stimuli than other cells of the insect body and have been widely used to assess the cyto-genotoxic effects of various xenobiotics^14^. γ-Terpinene showed significant cyto-genotoxic effects on the hemocytes of melon fly larvae. One of the probable causes for cyto-genotoxic effects can be due to generation of ROS by γ-terpinene treatment. ROS are generated as a part of normal metabolism of the cell. They can also be exogenously generated after exposure of animal to environmental stressors such as xenobiotics, and excessive levels of ROS can cause severe damage to biomolecules including DNA, proteins, lipids, organelles, and cellular membranes^71–73^. Damaged cells are targeted to be eliminated by various cell death pathways such as apoptosis. γ-Terpinene treatment also led to an increase in the frequency of apoptotic and necrotic cells in melon fruit fly larvae. Plant-based chemicals have been previously reported to show induction of apoptosis in insect immune cells. Çelik et al.^74^ have reported a decrease in viable hemocytes of lesser wax moth, *Achroia grisella* Fabricius at all treated doses of indole-3-acetic acid. An increase in apoptotic and necrotic cells was also reported by Altuntaş et al.^62^ in greater wax moth, *Galleria mellonella* (Linnaeus) larvae after treatment with various doses of tetracyclic di-terpenoid, gibberellic acid. The cytotoxic effects of γ-terpinene can also be one of the reasons for reduced number of circulating hemocytes in the hemolymph of Z. *cucurbitae* larvae.

Our results for genotoxic effects are supported by the findings of Dua et al.^75^. They too reported genotoxic effects of terpene rich essential oils of *Psoralea corylifolia* L. against southern house mosquito, *Culex quinquefasciatus* Say*.* Similar genotoxic effects were also reported by Attaullah et al.^76^, where they found an increase in tail length, % tail DNA, and tail moment in larvae of house fly, *Musca domestica* Linnaeus when exposed to plant extracts of *Peganum harmala* L., *Datura stramonium* L., and *Azadirachta indica* A. Juss. Prabu et al.^77^ also reported increase in tail length, % tail DNA, tail moment, and olive tail moment of DNA of hemocytes of rice moth, *Corcyra cephalonica* when treated with LC_50_ concentration of *Origanum majorana* L. essential oil rich in monoterpenes, cis-β-terpineol and terpinene-4-ol. Similarly, *S. litura* larvae when exposed to different concentrations of sesquiterpene, β-caryophyllene showed an increase in all comet parameters, tail length, % tail DNA, tail moment, and olive tail moment^7^.

## Methods

### Insect culture

*Z. cucurbitae* culture was maintained in the insect culture laboratory of the department. The rearing of fruit flies was done as suggested by Gupta et al.^78^. Fruits and vegetables infested with larvae were procured from local vegetable markets of Amritsar. After emergence, the adults were identified on the basis of keys given by White and Elson-Harris^36^ and Kapoor^79^. Adult flies were reared in wooden wire mesh cages under controlled conditions i.e., temperature of 25 ± 2 °C, 70–80% relative humidity, and a photoperiod of 10L:14D h. Flies were fed 20–25% sugar solution and protein X and oviposited on freshly cut pumpkin (*Cucurbita moschata* Duchesne) pieces. After oviposition by the female adults, the pumpkin pieces were transferred to plastic jars containing sterilized moist sand and covered with muslin cloth. On emergence, the adults were again transferred to wooden cages.

### Chemical used

γ-Terpinene (97% purity) was purchased from Sigma Aldrich Pvt. Ltd., India.

### Bioassays

For conducting bioassays, larvae were reared on artificial diet prepared by the methodology given by Srivastava^80^^.^ All the three larval instars i.e., first instar (44–48 h old), second instar (64–72 h old), and third instar (88–96 h old), after they had been harvested from pumpkin pieces, were transferred to sterilized vials containing artificial diet having different concentrations of γ-terpinene viz. 5, 25, 125, 625, and 3125 ppm. Artificial diet without γ-terpinene was taken as control. Various parameters such as percentage pupation, larval period, pupal period, total development period, and adult emergence were assessed for all the three larval instars. For each concentration, fifteen larvae were added to a replicate and a total of six replicates were taken.

### Growth indices

Formulae given by Kumar et al.^81^ was used to obtain the larval growth index (LGI) and total growth index (TGI).

$$\mathrm{LGI}=\mathrm{Percentage\,pupation}/\mathrm{Larval\,period}$$

$$\mathrm{TGI}=\mathrm{Percentage\,emergence}/\mathrm{Total\,development\,period}$$

### Pupal weight

Second instar (64–72 h old) larvae were fed on various concentrations of γ-terpinene i.e., 5, 25, 125, 625, and 3125 ppm along with control. Weight of the pupae was taken after larvae that had fed on control and treated diets metamorphosed into pupae. For each concentration, six replications were taken.

### Nutritional assays

To evaluate the effect of γ-terpinene on nutritional physiology of *Z. cucurbitae*, second instar larvae (64–72 h old) were used. They were fed on artificial diet containing different concentrations (5, 25, 125, 625, and 3125 ppm) of γ-terpinene along with control. Weight of larvae was measured before transferring to vials having artificial diet and after 48 h of feeding on diet incorporated with γ-terpinene. For each concentration, six replications were taken. Food assimilated (FA) and mean relative growth rate (MRGR) were calculated as described by Khan and Saxena^82^ and Martinez and Emden^83^, respectively.

$$\mathrm{FA }(\mathrm{mg})=\mathrm{Ti}\times \frac{\mathrm{Cf}-\mathrm{Ci}}{\mathrm{Ci}}+\mathrm{Tf}-\mathrm{Ti}$$

where, Ci = initial weight of control larvae, Cf = final weight of control larvae, Ti = initial weight of treated larvae, Tf = final weight of treated larvae.

$$\mathrm{MRGR }(\mathrm{mg}/\mathrm{mg}/\mathrm{day})=\frac{\mathrm{logN\,Final\,weight }\left(\mathrm{mg}\right)-\mathrm{logN\,Initial\,weight }(\mathrm{mg})}{\mathrm{Time}(\mathrm{in\,days})}$$

### Immunological studies

To conduct the immunological studies, second instar (64–72 h old) larvae were fed on artificial diet containing LC_30_ and LC_50_ concentrations of γ-terpinene as well as control diet. After feeding, the larvae at different time intervals viz. 24 h, 48 h, and 72 h were extracted for analyzing various immunological parameters.

#### Phenoloxidase enzyme assay

The methodology given by Zimmer^84^ was used to estimate phenoloxidase activity. Larvae were homogenized in 0.05 M potassium sodium phosphate buffer (pH 6.2) to prepare 1% homogenate (w/v). Assay mixture consisted of 300 µl of enzyme extract and 700 µl of catechol (50 mM, pH 6.2, and prepared in potassium sodium phosphate buffer). Absorbance was taken at 340 nm for 10 min at an interval of 1 min.

#### Total hemocyte count (THC)

The hemolymph was collected from ten larvae by chopping off their heads and was pooled. It was diluted with Tauber-Yeager fluid consisting of 4.65 g NaCl, 0.15 g KCl, 0.11 g CaCl_2_, 0.005 g Gentian violet, 0.125 ml Acetic acid, and 100 ml distilled water^85^. A drop of this diluted hemolymph was placed on Neubauer hemocytometer and covered with a coverslip. Hemocytes were observed under EVOS XL Core microscope at 20X magnification and cells were counted in outer four corner squares (1 mm square) of hemocytometer. For each treatment interval, six replications were taken. Formula given by Jones^86^ was used to calculate the number of circulating hemocytes per mm^3^.

$$\mathrm{THC}/{\mathrm{mm}}^{3}= \frac{\mathrm{Hemocytes\,in\,four\,}1\,\mathrm{ mm\,squares }\times \mathrm{ Dilution }\times \mathrm{ Depth\,factor\,of\,the\,chamber }}{\mathrm{Number\,of\,squares\,counted}}$$

where, Dilution = 2 times, Depth factor of the chamber = 10 (constant), number of squares counted = 4.

#### Differential hemocyte count (DHC)

Staining of hemocytes was done according to the methodology of Arnold and Hinks^87^ with slight modifications. Hemolymph was bled directly on a clean glass slide and a thin smear was made immediately. Slides were air dried for 20–30 min and afterward were fixed in methanol for 15–20 min. Staining was done for 10 min using Giemsa stain. Slides were then washed with distilled water and air dried. Hemocytes were observed under EVOS XL Core microscope at 40X magnification. A total of six replicates were taken for each treatment interval and in each replicate, 200 cells were counted.

### Cell viability assay

Acridine orange (AO)/Ethidium bromide (EtBr) double staining was used to check the viability of hemocytes. Viable, apoptotic, and necrotic cells were identified according to Altuntaş et al.^62^. Second instar (64–72 h old) larvae were fed on control and treated (LC_30_ and LC_50_) diets for 24 h, 48 h, and 72 h. Hemolymph was pooled from ten larvae and 5 µl of hemolymph was mixed with 10 µl of AO/EtBr dye mixture (consisting of 100 µg/ml of acridine orange and 100 µg/ml of ethidium bromide dissolved in PBS), transferred to a clean glass slide, covered with coverslip, and observed under Nikon ECLIPSE E200 fluorescent microscope at a magnification of 40X. Photographs were taken with Nikon D5300 camera. For each treatment interval, six replicates were taken and, in each replicate, 300 cells were counted.

### Comet assay

Single cell gel electrophoresis (comet assay) was conducted on larval hemocytes according to the methodology of Singh et al.^88^ with slight modifications to assess the genotoxic effects of γ-terpinene on melon fly.

#### Sample preparation

Second instar (64–72 h old) larvae were fed on control and γ-terpinene (LC_30_ and LC_50_) treated diets. After 24 h, 48 h, and 72 h of feeding, hemolymph was extracted from the larvae by chopping off the heads of larvae with the help of a sterile and sharp blade. Hemolymph of ten larvae was pooled and 10 µl of hemolymph was right away transferred to 40 µl of PBS having pH 7.4.

#### Buffers

##### Lysis buffer

Stock solution of lysis buffer (445 ml, pH 10) was prepared by dissolving 73.01 g of NaCl, 18.7 g EDTA, 0.6 g Tris, and 4 g NaOH in distilled water. Just before dipping the slides in lysis buffer, a working solution of lysis buffer was prepared by adding 44.5 ml of DMSO and 4.95 ml of Triton X to the lysis stock.

##### Electrophoresis buffer

Two separate stock solutions of NaOH (40 g of NaOH in 100 ml double distilled water) and EDTA (7.44 g of EDTA in 100 ml of double distilled water) were prepared. Just before conducting electrophoresis, a working electrophoresis buffer was prepared by adding 30 ml of NaOH stock and 5 ml of EDTA stock to 965 ml of double distilled water (chilled).

##### Tris buffer

Tris buffer (4.84 g) was dissolved in 100 ml of double distilled water to prepare Tris buffer (pH 7.4).

#### Preparation of slide and electrophoresis

Normal melting point agarose (NMPA) (1%) was applied on the glass slide as base layer 12 h prior to the hemolymph sample layering. Hemolymph sample (35 µl) was mixed with 110 µl of 0.5% low melting point agarose (LMPA) and layered on top of the base layer. Slides were then covered with coverslips and kept in refrigerator (4 °C) for 15–20 min. After this, the coverslips were removed and another layer of 0.5% LMPA was layered on the slide. Slides were again kept in the refrigerator. To lyse the cells, slides were placed in lysis buffer for 2–3 h, at 4 °C, and in a dark place. Slides were removed from the lysis buffer after 2–3 h and placed on a horizontal electrophoretic unit. The unit was then filled with chilled electrophoresis buffer and slides were left dipped in electrophoresis buffer for 20 min. After dipping, electrophoresis was conducted at 300 mA and 20 V for 20 min. After culmination of the electrophoresis, slides were removed from the unit and neutralized with Tris buffer and a final wash of chilled distilled water was given to the slides.

#### Staining and analysis

Staining of the slides was done with ethidium bromide (EtBr). Slides were covered with coverslips and observed under Nikon ECLIPSE E200 fluorescent microscope at a magnification of 40X. Nikon D5300 camera was used to take photographs of the slides. Casp Lab software was used to measure comet parameters viz. tail length (µm), % tail DNA, tail moment (TM), and olive tail moment (OTM). For each treatment interval, 150 cells were analyzed.

### Statistical analysis

One-way ANOVA (analysis of variance) with Tukey’s test at p ≤ 0.05 was used to compare differences in mean. SPSS version 16.0 and Microsoft Excel were used to perform statistical analysis. Values are represented as Mean ± SE. The LC_30_ and LC_50_ values for second instar larvae were computed using regression equation in MS Excel 2019.

## Conclusion

The present study revealed growth regulatory effects of γ-terpinene on *Z. cucurbitae* evident from delayed development of the larvae, decreased pupation and adult emergence, and reduced pupal weight. Moreover, the pupae and adults formed were deformed. The decline in pupal weight and deformities in pupae and adults could adversely affect the reproduction and fertility rate of the insect leading to its diminished population build-up. The nutritional physiology of *Z. cucurbitae* was also affected which could be due to metabolic cost incurred to counter the toxicity caused by γ-terpinene. Low levels of phenoloxidase and decline in the total hemocyte count indicated that the immune system of the insect was compromised. An immunologically compromised pest will have reduced chances of survival in natural agroecosystems being more prone to secondary infections by other biocontrol agents. Furthermore, γ-terpinene also exerted cytotoxic and genotoxic effects on hemocytes of *Z. cucurbitae* and as hemocytes are multifunctional in nature, any damage to hemocytes can affect various vital processes inside an insect’s body. The findings of the present study may pave the way for developing novel means for regulating pest populations by strategically disrupting the normal developmental processes and weakening the insect immune defense and integrity of the genome.

## Data Availability

Data sets used or analyzed during current study are available from the corresponding author on reasonable request.

## References

[1] Sarwar M (2016). Potential uses of synergists in insecticides resistance management accompanied by their contributions as control agents and research tools. Chem. Res. J..

[2] Chen Y (2019). Ecological risk assessment of the increasing use of the neonicotinoid insecticides along the east coast of China. Environ. Int..

[3] Anaya-Gil J, Ramos-Morales P, Muñoz-Hernandez A, Bermúdez A, Gomez-Estrada H (2022). In vivo evaluation of the toxic activity and genotoxicity of the Hymenaea courbaril L’s resin in Drosophila melanogaster. Saudi J. Biol. Sci..

[4] Razaq M, Shah FM, Rakshit A (2022). Biopesticides for management of arthropod pests and weeds. Advances in Bio-inoculant Science, Biopesticides.

[5] Benelli G, Pavela R, Maggi F, Petrelli R, Nicoletti M (2017). Commentary: making green pesticides greener? The potential of plant products for nanosynthesis and pest control. J. Clust. Sci..

[6] Singh S, Diksha Mahajan E, Sohal SK (2022). Appraisal of growth inhibitory, biochemical and genotoxic effects of Allyl Isothiocyanate on different developmental stages of *Zeugodacus cucurbitae* (Coquillett) (Diptera: Tephritidae). Sci. Rep..

[7] Mahajan E, Singh S, Diksha Kaur, S. & Sohal, S.K. (2022). The genotoxic, cytotoxic and growth regulatory effects of plant secondary metabolite β-caryophyllene on polyphagous pest *Spodoptera litura* (Fabricius) (Lepidoptera: Noctuidae). Toxicon.

[8] Kaur M, Kumar R, Upendrabhai DP, Singh IP, Kaur S (2017). Impact of sesquiterpenes from *Inula racemosa* (Asteraceae) on growth, development and nutrition of *Spodoptera litura* (Lepidoptera: Noctuidae). Pest Manag. Sci..

[9] Datta R, Kaur A, Saraf I, Singh IP, Kaur S (2019). Effect of crude extracts and purified compounds of *Alpinia galanga* on nutritional physiology of a polyphagous lepidopteran pest, *Spodoptera litura* (Fabricius). Ecotoxicol. Environ. Saf..

[10] Ahmad S (1992). Biochemical defence of pro-oxidant plant allelochemicals by herbivorous insects. Biochem. Syst. Ecol..

[11] González-Santoyo I, Córdoba-Aguilar A (2012). Phenoloxidase: A key component of the insect immune system. Entomol. Exp. Appl..

[12] Lavine MD, Strand MR (2002). Insect hemocytes and their role in immunity. Insect Biochem. Mol. Biol..

[13] Sharma PR, Sharma OP, Saxena BP (2008). Effect of sweet flag rhizome oil (*Acorus calamus*) on hemogram and ultrastructure of hemocytes of the tobacco armyworm, *Spodoptera litura* (Lepidoptera: Noctuidae). Micron.

[14] Kaur HP, Singh B, Thakur A, Kaur A, Kaur S (2015). Studies on immunomodulatory effect of endophytic fungus *Alternaria alternata* on *Spodoptera litura*. J. Asia Pac. Entomol..

[15] Kiger JA, Natzle JE, Green MM (2001). Hemocytes are essential for wing maturation in *Drosophila melanogaster*. Proc. Natl. Acad. Sci. USA.

[16] De Sousa DP (2011). Analgesic-like activity of essential oils constituents. Molecules.

[17] Abdelgaleil SAM, Gad HA, Ramadan GR, El-Bakry AM, El-Sabrout AM (2021). Monoterpenes: chemistry, insecticidal activity against stored product insects and modes of action: A review. Int. J. Pest Manag..

[18] Moreira RC, Vespermann KA, Molina G, Bicas JL, Marostica Junior MR, Gupta VK (2022). Health properties of dietary monoterpenes. Biomolecules from Natural Sources: Advances and Applications.

[19] Vranová E, Coman D, Gruissem W (2013). Network analysis of the MVA and MEP pathways for isoprenoid synthesis. Annu. Rev. Plant Biol..

[20] Liu L (2021). Integrating RNA-seq with functional expression to analyze the regulation and characterization of genes involved in monoterpenoid biosynthesis in *Nepeta tenuifolia* Briq. Plant Physiol. Biochem..

[21] Faria JMS, Rusinque L, Vicente CSL, Inácio ML (2022). Bioactivity of monoterpene alcohols as an indicator of biopesticidal essential oils against the root knot nematode *Meloidogyne ethiopica*. Biol. Life Sci. Forum.

[22] Borugă O (2014). Thymus *vulgaris* essential oil: Chemical composition and antimicrobial activity. J. Med. Life.

[23] Channoo C, Tantakom S, Jiwajinda S, Isichaikul S (2002). Fumigation toxicity of eucalyptus oil against three stored-product beetles. Thai J. Agric. Sci..

[24] Jalali-Heravi M, Zekavat B, Sereshti H (2007). Use of gas chromatography–mass spectrometry combined with resolution methods to characterize the essential oil components of Iranian cumin and caraway. J. Chromatogr. A.

[25] Abbassy MA, Abdelgaleil SAM, Rabie RY (2009). Insecticidal and synergistic effects of *Majorana hortensis* essential oil and some of its major constituents. Entomol. Exp. Appl..

[26] Passos FF (2015). Involvement of cholinergic and opioid system in γ-terpinene-mediated antinociception. Evid. Based Complement. Alter. Med..

[27] Ebadollahi A, Ashrafi Parchin R, Farjaminezhad M (2016). Phytochemistry, toxicity and feeding inhibitory activity of Melissa officinalis L essential oil against a cosmopolitan insect pest,* Tribolium** castaneum* Herbst. Toxin Rev..

[28] Cetin H (2010). Acaricidal activity of* Satureja** thymbra* L. essential oil and its major components, carvacrol and γ-terpinene against adult Hyalomma marginatum (Acari: Ixodidae). Vet. Parasitol..

[29] Tahvilian R (2016). Ethnomedicinal plants: Study on antifungal activity of essential oil of *Pistacia khinjuk* (combined with the dominance γ-terpinene) against *Candida albicans*. Int. J. Pharm. Clin. Res..

[30] Sousa LG (2022). Synergistic effects of carvacrol, α-terpinene, γ-terpinene, ρ-cymene and linalool against *Gardnerella* species. Sci. Rep..

[31] Zochedh A, Priya M, Shunmuganarayanan A, Thandavarayan K, Sultan AB (2022). Investigation on structural, spectroscopic, DFT, biological activity and molecular docking simulation of essential oil Gamma-Terpinene. J. Mol. Struct..

[32] Nooshadokht M (2022). In silico and in vitro antileishmanial effects of gamma-terpinene: Multifunctional modes of action. Chem. Biol. Int..

[33] Guo Y, Baschieri A, Amorati R, Valgimigli L (2021). Synergic antioxidant activity of γ-terpinene with phenols and polyphenols enabled by hydroperoxyl radicals. Food Chem..

[34] Astani A, Reichling J, Schnitzler P (2010). Comparative study on the antiviral activity of selected monoterpenes derived from essential oils. Phytother. Res..

[35] Gong X, Ren Y (2020). Larvicidal and ovicidal activity of carvacrol, p-cymene, and γ-terpinene from *Origanum vulgare* essential oil against the cotton bollworm, *Helicoverpa armigera* (Hübner). Environ. Sci. Pollut. Res..

[36] White IM, Elson-Harris MM (1992). Fruit Flies of Economic Significance: Their Identification and Bionomics.

[37] Dhillon MK, Singh R, Naresh JS, Sharma HC (2005). The melon fruit fly, *Bactrocera cucurbitae*: A review of its biology and management. J. Insect Sci..

[38] Wu Y (2011). Microsatellite markers reveal population structure and low gene flow among collections of *Bactrocera cucurbitae* (Diptera: Tephritidae) in Asia. J. Econom. Entomol..

[39] Koyama J, Kakinohana H, Miyatake T (2004). Eradication of the melon fly, *Bactrocera cucurbitae*, in Japan: Importance of behavior, ecology, genetics, and evolution. Annu. Rev. Entomol..

[40] Benelli G, Flamini G, Canale A, Cioni PL, Conti B (2012). Toxicity of some essential oil formulations against the Mediterranean fruit fly *Ceratitis capitata* (Wiedemann) (Diptera Tephritidae). Crop Prot..

[41] Benelli G (2013). Biotoxicity of *Melaleuca alternifolia* (Myrtaceae) essential oil against the Mediterranean fruit fly, *Ceratitis capitata* (Diptera: Tephritidae), and its parasitoid *Psyttalia concolor* (Hymenoptera: Braconidae). Ind. Crops Prod..

[42] Canale A (2013). Ingestion toxicity of three Lamiaceae essential oils incorporated in protein baits against the olive fruit fly, *Bactrocera oleae* (Rossi) (Diptera Tephritidae). Nat. Prod. Res..

[43] Ruiz MJ (2014). Toxic effect of citrus peel constituents on *Anastrepha fraterculus* Wiedemann and *Ceratitis capitata* Wiedemann immature stages. J. Agric. Food Chem..

[44] El-Minshawy AM, Abdelgaleil SAM, Gadelhak GG, Al-Eryan MA, Rabab RA (2018). Effects of monoterpenes on mortality, growth, fecundity, and ovarian development of *Bactrocera zonata* (Saunders) (Diptera: Tephritidae). Environ. Sci. Poll. Res..

[45] Abdelgaleil SAM, Al-Eryan MA, El-Minshawy A, Gadelhak GG, Rabab RA (2019). Toxicity, developmental and histological effects of monoterpenes on peach fruit fly, *Bactrocera zonata* (Diptera: Tephritidae). J. Crop Prot..

[46] Rizzo R (2020). Developing green insecticides to manage olive fruit flies? Ingestion toxicity of four essential oils in protein baits on *Bactrocera oleae*. Ind. Crops Prod..

[47] Jaffar S, Lu Y (2022). Toxicity of some essential oils constituents against oriental fruit fly, *Bactrocera dorsalis* (Hendel) (Diptera: Tephritidae). Insects.

[48] Ouarhach A (2022). Evaluation of insecticidal activity of *Lavandula coronopifolia* essential oil against the Mediterranean fruit fly *Ceratitis capitata* Wiedemann (Diptera: Tephritidae). S. Afr. J. Bot..

[49] Gupta A, Gupta A (1979). Hemocyte types: Their structures, synonymies, interrelationships, and taxonomic significance. Insect Hemocytes: Development, Forms, Functions and Techniques.

[50] Jiang Z, Akhtar Y, Bradbury R, Zhang X, Isman MB (2009). Comparative toxicity of essential oils of* Litsea pungens* and Litsea cubeba and blends of their major constituents against the cabbage looper,* Trichoplusia** ni*. J. Agric. Food Chem..

[51] Wang Y (2015). Bioactivity of essential oil of *Zingiber purpureum* rhizomes and its main compounds against two stored product insects. J. Econom. Entomol..

[52] Erler F (2005). Fumigant activity of six monoterpenoids from aromatic plants in Turkey against the two stored-product pests confused flour beetle, Tribolium confusum, and Mediterranean flour moth,* Ephestia** kuehniella*. J. Plant Dis. Prot..

[53] Basij M, Sahebzadeh N, Shahriari M, Panahandeh S (2023). Insecticidal potential of Ajwain essential oil and its major components against *Chilo suppressalis* Walker. J. Plant Dis. Prot..

[54] Abdelgaleil SAM, Al-Nagar N, Abou-Taleb HK, Shawir MS (2022). Effect of monoterpenes, phenylpropenes and sesquiterpenes on development, fecundity and fertility of *Spodoptera littoralis* (Boisduval). Int. J. Trop. Insect Sci..

[55] Abdelgaleil SAM, Abou-Taleb HK, Al-Nagar N, Shawir MS (2020). Antifeedant, growth regulatory and biochemical effects of terpenes and phenylpropenes on *Spodoptera littoralis* Boisduval. Int. J. Trop. Insect Sci..

[56] Ismail SM, Hassan NA, Wahba TF, Shaker N (2022). Chemical composition and bioactivities of *Melaleuca alternufolia* essential oil and its main constituents against *Spodoptera littoralis* (Boisaduval, 1833). Bull. Natl. Res. Cent..

[57] Chen YZ (2021). Detoxification, antioxidant, and digestive enzyme activities and gene expression analysis of *Lymantria dispar* larvae under carvacrol. J. Asia Pac. Entomol..

[58] Agliassa C, Maffei ME (2018). *Origanum vulgare* terpenoids induce oxidative stress and reduce the feeding activity of *Spodoptera littoralis*. Int. J. Mol. Sci..

[59] Pintong AR (2020). Insecticidal and histopathological effects of Ageratum conyzoides weed extracts against dengue vector,* Aedes** aegypti*. Insects.

[60] Karthi S (2018). Effect of Aspergillus flavus on the mortality and activity of antioxidant enzymes of *Spodoptera*
*litura* Fab. (Lepidoptera: Noctuidae) larvae. Pestic. Biochem. Physiol..

[61] Zhiqing M, Xiuling H, Juntao F (2008). Effects of terpinen-4-ol on 4 kinds of metabolizing enzymes and PPO in *Mythimna separta* Walker. Sci. Agric. Sin..

[62] Altuntaş H, Kılıç AY, Uçkan F, Ergin E (2012). Effects of gibberellic acid on hemocytes of *Galleria*
*mellonella* L. (Lepidoptera: Pyralidae). Environ. Entomol..

[63] Zibaee A, Bandani AR (2010). Effects of *Artemisia annua* L. (Asteracea) on the digestive enzymatic profiles and the cellular immune reactions of the Sunn pest*, **Eurygaster*
*integriceps* (Heteroptera: Scutellaridae), against *Beauveria*
*bassiana*. Bull. Entomol. Res..

[64] Er A, Keskin M (2016). Influence of abscisic acid on the biology and hemocytes of the model insect *Galleria mellonella* (Lepidoptera: Pyralidae). Ann. Entomol. Soc. Am..

[65] Ali AM, Ibrahim AM (2018). Castor and camphor essential oils alter hemocyte populations and induce biochemical changes in larvae of *Spodoptera littoralis* (Boisduval) (Lepidoptera: Noctuidae). J. Asia Pac. Entomol..

[66] Sadeghi R, Eshrati MR, Mortazavian SM, Jamshidnia A (2019). The Effects of the essential oils isolated from four ecotypes of cumin (*Cuminum*
*cyminum* L.) on the blood cells of the pink stem borer, *Sesamia*
*cretica* Ledere (Lepidoptera: Noctuidae). J. Kans. Entomol. Soc..

[67] Dorrah MA, Mohamed AA, Shaurub ES (2019). Immunosuppressive effects of the limonoid azadirachtin, insights on a nongenotoxic stress botanical, in flesh flies. Pestic. Biochem. Physiol..

[68] Afraze Z, Sendi JJ (2021). Immunological and oxidative responses of the lesser mulberry pyralid, *Glyphodes pyloalis* by an aqueous extract of *Artemisia annua* L. Invertebr. Surviv. J..

[69] Pandey S, Pandey JP, Tiwari RK (2012). Effect of some botanicals on hemocytes and molting of *Papilio demoleus* larvae. J. Entomol..

[70] Afraze Z, Sendi JJ, Karimi-Malati A, Zibaee A (2020). Methanolic extract of winter cherry causes morpho-histological and immunological ailments in mulberry pyralid *Glyphodes pyloalis*. Front. Physiol..

[71] Iwański B, Mizerska-Kowalska M, Andrejko M (2023). *Pseudomonas aeruginosa* exotoxin A induces apoptosis in *Galleria mellonella* hemocytes. J. Invertebr. Pathol..

[72] Redza-Dutordoir M, Averill-Bates DA (2016). Activation of apoptosis signaling pathways by reactive oxygen species. Biochim. Biophys. Acta.

[73] Fujii J, Soma Y, Matsuda Y (2023). Biological action of singlet molecular oxygen from the standpoint of cell signaling, injury and death. Molecules.

[74] Çelik D, Özbek R, Uçkan F (2017). Effects of indole-3-acetic acid on hemocytes of Achoria grisella Fabr. (Lepidoptera: Pyralidae). J. Entomol. Res. Soc..

[75] Dua VK, Kumar A, Pandey AC, Kumar S (2013). Insecticidal and genotoxic activity of *Psoralea*
*corylifolia *Linn. (Fabaceae) against Culex quinquefasciatus Say, 1823. Parasites Vectors.

[76] Attaullah (2020). Insecticidal, biological and biochemical response of *Musca domestica* (Diptera: Muscidae) to some indigenous weed plant extracts. Saudi J. Biol. Sci..

[77] Prabu S, Jing D, Chandran V, Mathew P (2020). Insecticidal activity of *Origanum*
*majorana* L. essential oil as anti-cholinergic agent. Entomol. Res..

[78] Gupta JN, Verma AN, Kashyap RK (1978). An improved method for mass rearing for melon fruit fly *Dacus cucurbitae* Coquillett. Indian J. Entomol..

[79] Kapoor VC, Kapoor VC (1993). Economic fruit flies. Indian fruit flies (Insecta: Diptera: Tephritidae).

[80] Srivastava BG (1975). A chemically defined diet for *Dacus*
*cucurbitae* (Coq.) larvae under aseptic conditions. Entomol. News Lett..

[81] Kumar A, Sood S, Mehta V, Nadda G, Shanker A (2004). Biology of *Thysanoplusia*
*orichalcea* (Fab.) in relation to host preference and suitability for insect culture and bioefficacy. Indian J. Appl. Entomol..

[82] Khan ZR, Saxena RC (1985). Behavioural and physiological responses of *Sogatella furcifera* (Homoptera: Delphacidae) to selected resistant and susceptible rice cultivars. J. Econom. Entomol..

[83] Martinez SS, Emden HFV (2001). Growth disruption, abnormalities and mortality of *Spodoptera littoralis* (Boisduval) (Lepidoptera: Noctuidae) caused by azadirachtin. Neotrop. Entomol..

[84] Zimmer M, Bärlocher F, Gessner M, Graça M (2020). Phenol oxidation. Methods to Study Litter Decomposition.

[85] Tauber OE, Yeager JF (1935). On total hemolymph (Blood) cell counts of insects I. Orthoptera, odonata, hemiptera, and homoptera. Ann. Entomol. Soc. Am..

[86] Jones JC (1962). Current concepts concerning insect hemocytes. Am. Zool..

[87] Arnold J, Hinks C, Gupta A (1979). Insect hemocytes under light microscopy: Techniques. Insect Hemocytes: Development, Forms, Functions and Techniques.

[88] Singh NP, McCoy MT, Tice RR, Schneider EL (1988). A simple technique for quantitation of low levels of DNA damage in individual cells. Exp. Cell Res..

